# Dexmedetomidine prevent postoperative nausea and vomiting on patients during general anesthesia: A PRISMA-compliant meta analysis of randomized controlled trials

**DOI:** 10.1097/MD.0000000000005770

**Published:** 2017-01-10

**Authors:** Shenhui Jin, Dong Dong Liang, Chengyu Chen, Minyuan Zhang, Junlu Wang

**Affiliations:** Department of Anesthesiology, The first Affiliated Hospital of Wenzhou Medical University, Wenzhou, China.

**Keywords:** dexmedetomidine, meta-analysis, nausea, vomiting

## Abstract

**Background:**

Postoperative nausea and vomiting (PONV) is a frequent complication in postoperative period. The aim of this article was to evaluate the effect of dexmedetomidine on PONV.

**Method:**

RevMan 5.3 software was applied for performing statistic analysis. Twenty-four trials with 2046 patients were included.

**Results:**

The PONV of the dexmedetomidine group was significantly lower compared with the placebo group (0.56, 95% CI: 0.46, 0.69). Subgroup analysis further confirmed the effect of dexmedetomidine (irrespective of administration mode) (*P* < 0.00001). Perioperative fentanyl consumption in dexmedetomidine group were also reduced significantly (*P* < 0.00001). Whereas, side effects such as bradycardia, hypotension increased in dexmedetomidine group (especially in loading dose mode and loading dose plus continuous infusion mode).

**Conclusions:**

Dexmedetomidine administrated in continuous infusion mode has the advantage to prevent PONV as well as reduce side effects such as bradycardia and hypotension.

## Abstract 2

Supplemental Digital Content is available in the text

## 1 Introduction

General anesthesia is widely used in several surgeries. It can cause some complications such as postoperative nausea and vomiting (PONV) and cognitive dysfunction. PONV is more common in general anesthesia than spinal anesthesia.^[1,2]^ Also, it can cause electrolyte imbalance and aggravate bleeding that delay hospital discharge.^[3]^ It is reported that PONV is even higher especially after gynecologic surgery, ranging from 24% to 75%, even up to 90%.^[4]^ Some clear risks including female gender, postoperative opioid treatment, the history of motion sickness and/or PONV and nonsmoker have been shown to independently predict PONV.^[5,6]^

Dexmedetomidine is a potent and highly selective α2-adrenoceptor agonist, which binds to transmembrane G protein-binding receptor located in the brain and spinal cord. It affects the functions of central nervous, circulatory systems and exhibits sedative, analgesic, sympatholytic properties.^[7]^ It has been widely used in different clinical settings like department of anesthesiology and intensive care unit (ICU).^[8]^ Recently, the effect of dexmedetomidine on PONV has been the focus of clinical researchers. Nevertheless, controversy about the effectiveness of dexmedetomidine for PONV is still ongoing, for different results reported in associated literature.

To our knowledge, there was no updated analysis done for combination of related data during general anesthesia. Therefore, we performed this meta-analysis to investigate the antiemetic effect of dexmedetomidine in patients undergoing general anesthesia.

## 2 Materials and methods

### 2.1 Ethical statement

All results and analyses were from previous published studies, thus no ethical approval and patient consent are required.

### 2.2 Search strategy

This meta-analysis were performed in accordance with recommendations of the Cochrane Handbook for Systematic Reviews of Interventions and was reported in compliance with the PRISMA (Preferred Reporting Items for Systematic Reviews and Meta-Analyses statement) guidelines.^[9]^ We performed a systematic electronic search in PubMed for relevant studies of randomized controlled trials (RCTs) published before August 2016. We used the following Medical Subject Heading (MeSH) terms and corresponding keywords dexmedetomidine, general anesthesia, and postoperative nausea and vomiting. Hand searching techniques also were used to identify appropriate studies. Moreover, articles that met the following criteria were included: randomized and double-blind study design; the intervention was treatment with dexmedetomidine given systemically in any dose during the perioperative period; patient undergone general anesthesia experiencing PONV.

### 2.3 Data extraction and analysis

All data were extracted by 2 reviewers (SH-Jin and DD-Liang) and then independently reviewing every selection for accuracy and consistency. Any discrepancy was resolved by JL-Wang for discussion and consensus. The following outcome measures were extracted from the retrieved reports: perioperative fentanyl consumption, number of patients experiencing PONV, number of patients undergoing bradycardia or hypotension. Moreover, the subgroup analysis was performed for different dexmedetomidine administration modes.

The following data were also collected by S-H. Jing and confirmed by other authors (CY Chen and MY Zhang): first author, year of publication, participants, type of surgery, administration mode of dexmedetomidine, comparisons, number of patients. Extracted data were entered into a standardized Excel (Microsoft Corporation, The Redmond, Washington, US) file.

### 2.4 Risk of bias assessment

**Figure 1:**
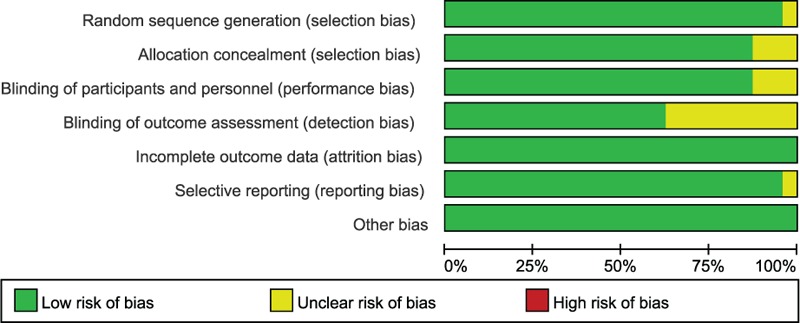
The risk of bias of included studies.

The risk of bias of included studies was assessed independently by 2 authors (SH-Jin and CY Chen) using the Cochrane risk-of-bias tool. We reviewed each trial and scored as “high,” “low,” or “unclear” risk of bias to the following criteria: random sequence generation; allocation concealment; blinding of participants and personnel; blinding of outcome assessment; incomplete outcome data; selective reporting; and other bias (Fig. 1).

### 2.5 Statistical analysis

Statistical analysis was performed using the Review Manager 5.3 software. We calculated relative risks (RRs) with 95% CIs for dichotomous outcomes by the Mantel–Haenszel method (fixed or random models). Continuous outcomes measured were expressed as a mean value and standard deviation and were analyzed by using weighted mean differences (WMD). *I*-square (*I*^2^) test was performed to assess the impact of study heterogeneity on the results of the meta-analysis. According to the Cochrane review guidelines, if severe heterogeneity was present at *I*^2^ > 50%, the random effect models were chosen, otherwise the fixed effect models were used. The funnel plot was used to detect potential publication bias.

## 3 Results

### 3.1 Trial selection

The process of literature screening, study selection, and reasons for exclusion was shown in the flow diagram. Our initial search yielded 102 records. After removing duplicates and screening the titles and abstracts, 24 RCTs published during 2005 to 2016 met the criteria and were included in the analysis.

### 3.2 Trials characteristics

The main characteristics of the included trials are summarized in Table 1.

**Table 1:**
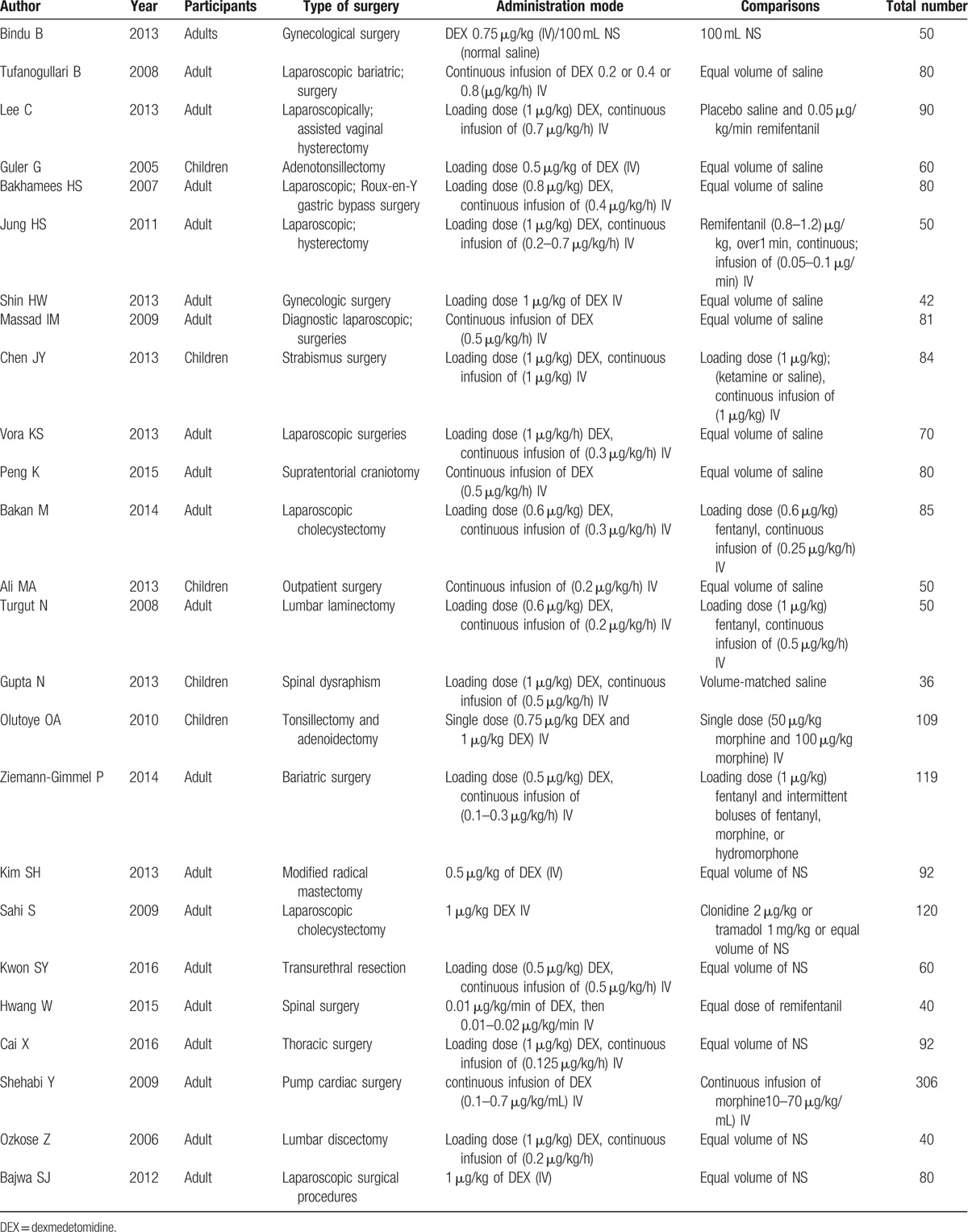
Characteristics of the included trials.

### 3.3 PONV

**Figure 2:**
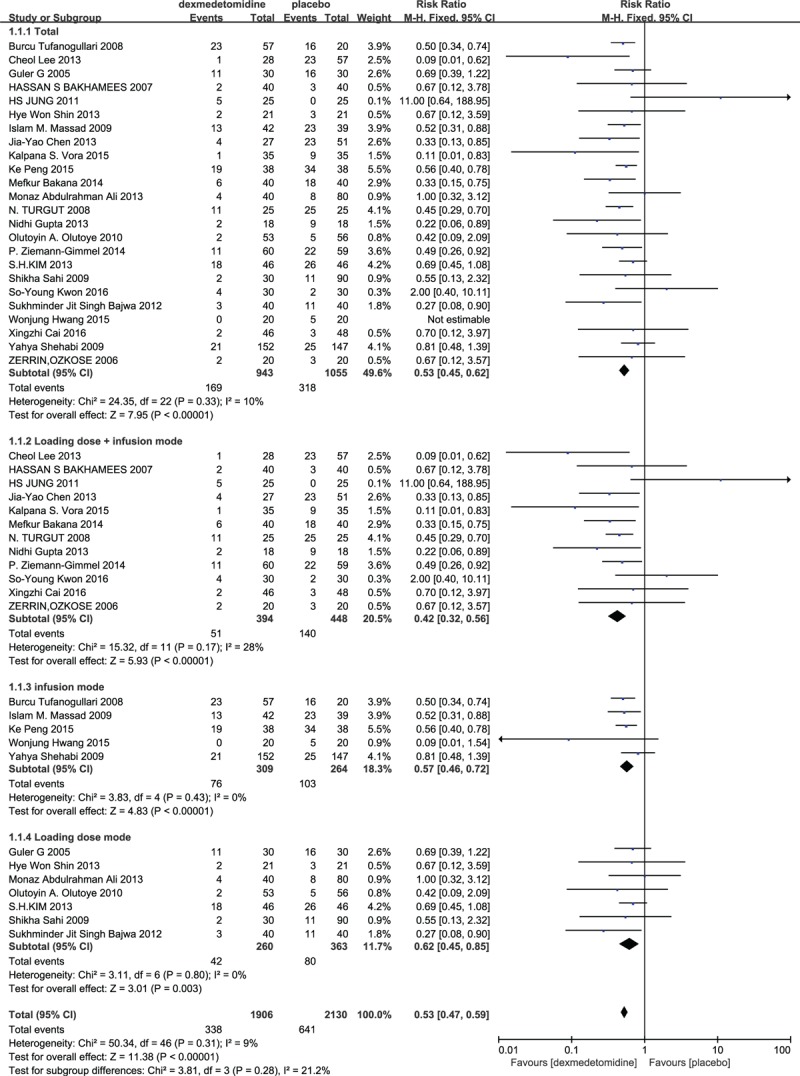
The total effect of dexmedetomidine and different infusion modes on PONV.

PONV was reported in 16 studies. As between-study heterogeneity not existed (*P* = 0.33), a fixed-effects model was adopted. The combined MD was 0.53 (95% CI: 0.45, 0.62) and it was significant (*Z* = 5.60, *P* < 0.00001).^[10–24]^ Thus the PONV of the dexmedetomidine group was significantly lower compared with the control group. Subgroup analysis showed that dexmedetomidine administration by loading dose plus continuous infusion or by loading dose or just by continuous infusion, the incidence of PONV during general anesthesia was decreased significantly (Fig. 2).^[10–34]^

#### 3.3.1 Perioperative fentanyl consumption

**Figure 3:**
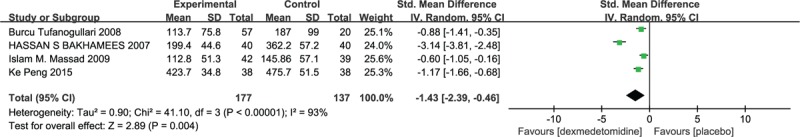
Perioperative fentanyl consumption in dexmedetomidine and placebo group.

Four trials assessed the need for use of perioperative fentanyl. The pooled analysis shown a significant decrease in need for the use of fentanyl (SMD Std. Mean Difference −1.43 (95% CI: −2.39, −0.46), although the study heterogeneity was high^[10,13,16,19]^ (Fig. 3).

#### 3.3.2 The effect of dexmedetomidine on children and adult

Five trials reported the effect of dexmedetomidine on children and 19 trials about adult. The pooled analysis shown total incidence of PONV was 13.69% in dexmedetomidine group. The combined MD was 0.50 (95% CI: 0.33, 0.76) and it was significant (*Z* = 3.22, *P* = 0.001). However, the total incidence of PONV in placebo group was 25.96%. The combined MD was 0.54 (95% CI: 0.45, 0.64) and it was significant (*Z* = 7.27, *P* < 0.00001) (Supplemental Fig).

### 3.4 Side effects

#### 3.4.1 Incidence of bradycardia

**Figure 4:**
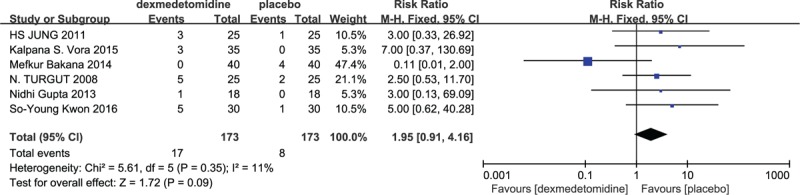
Incidence of bradycardia in dexmedetomidine and placebo group.

Six studies described the incidence of bradycardia.^[14,18,20,22,23,28]^ A fixed-effect model was adopted since no between-study heterogeneity was found (*P* > 0.05). The pooled RR was determined as 5.0 (95% CI: 1.70, 14.72) and it was no difference according to the statistical result (*Z* = 2.92, *P* = 0.09) (Fig. 4).

#### 3.4.2 Incidence of hypotension

**Figure 5:**
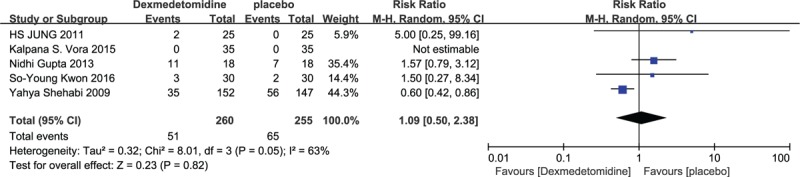
Incidence of hypotension in dexmedetomidine and placebo group.

There were 5 studies reporting perioperative hypotension.^[14,18,23,28,32]^ Compared with placebo, no difference was found between 2 groups (*P* = 0.82) (Fig. 5).

### 3.5 Risk of bias

**Figure 6:**
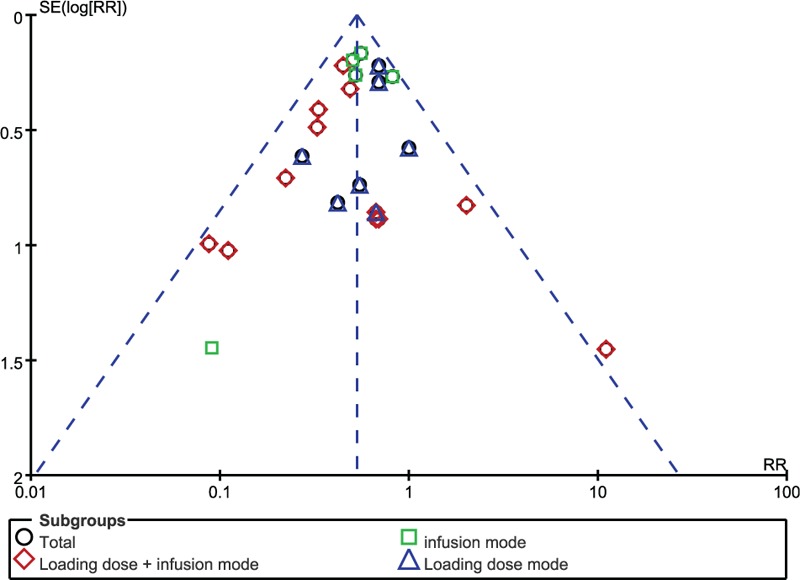
Test for publication bias of the studies included in the incidence of PONV. PONV = postoperative nausea and vomiting.

The funnel plot was applied for assessing publication bias of studies included in the incidence of PONV in this meta-analysis. No evident publication bias was obtained through the visual distribution (Fig. 6).

## 4 Discussion

Through this meta-analysis we found that: dexmedetomidine, regardless of administration modes (by loading dose or loading dose plus continuous infusion or just infusion) significantly reduced the incidence of PONV in adult or children, compared to placebo, administration of dexmedetomidine diminished the perioperative fentanyl consumption, however, dexmedetomidine increased adverse events such as bradycardia and hypotension in loading dose or loading dose plus continuous infusion mode, indicating that dexmedetomidine in continuous infusion mode is superiority to prevent PONV.

Several meta-analyses regarding this topic have been published.^[35,36]^ Although the main finding of our meta-analysis was consistent with previous meta-analyses. Differences between our meta-analysis and the previous ones should be noted. One meta-analysis included 15 trials with 899 patients and the other only included 11 trials with 692 patients, our present meta-analysis included 24 trials totaling 2046 patients with added statistical power of at least 1100 cases. Our present meta-analysis further reinforces earlier results of previous meta-analyses.

PONV is more common complication during general anesthesia than during spine anesthesia.^[1,37,38]^ In clinical setting, PONV are treated effectively by antiemetics such as ondansetron. However, patients may experience headache, dizziness as well as drowsiness/sedation when ondansetron is used, which limit its wide application. Dexmedetomidine, as an anesthetic adjunct for general and regional anesthesia, has been demonstrated to reduce PONV. Our meta-analysis reached the same conclusion showing that dexmedetomidine reduced PONV significantly. Intriguing, there is no study report whether dexmedetomidine is superior to antiemetic like ondansetron for treatment of PONV. Herein we supposed that antiemetics combination with dexmedetomidine have more advantages to treat PONV. The antiemetic effect may be induced by direct antiemetic properties of α2 agonists through inhibition of catecholamine by parasympathetic tone. Also, administration of dexmedetomidine reduced the perioperative fentanyl consumption in this study may explain the decreased incidence of PONV.

In order to distinguish the effect of different administration modes of dexmedetomidine, we performed subgroup analysis and further exhibited the antiemetic effect of dexmedetomidine. In this analysis, we found 7 articles using loading dose mode (0.5–1 μg/kg), 12 articles using loading dose (0.5–1 μg/kg) and continuous infusion (0.1–0.7 μg/kg/h) mode, and 5 articles by continuous infusion mode (0.1–0.7 μg/kg/h). Moreover, the higher incidence of hypotension was found in loading dose and continuous infusion mode in this analysis. One study demonstrated satisfactory hemodynamic effects when administered without a loading infusion at doses between 0.2 and 0.4 μg/kg/h.^[39]^ Therefore, many clinicians have decided to forego the administration of a loading dose. Based on the results from this report, we advocate to use continuous infusion mode of dexmedetomidine (0.1–0.7 μg/kg/h).

Dexmedetomidine has an onset of action after approximately 15 minutes and peaked at 1 hour after continuous infusion. Its distribution half-life (*t*½α) is 6 minutes in adults over the dose ranges of 0.2 to 0.7 μg/kg/h. While, its elimination half-life (*t*½β) range from 2.0 to 2.5 hours and a clearance of 39 L/h. The similar rates of infusion can be used in children and adults to produce a steady state plasma concentration.^[40,41]^ It can prevent surgical stress response by decreasing blood pressure and heart rate.^[42]^ Unfortunately, dexmedetomidine can cause hypotension and bradycardia in clinical, especially in patient with hypovolemia or atrioventricular block. In our study, the number of perioperative hypotension and bradycardia was increased in patients with dexmedetomidine, although no statistical significance was found between dexmedetomidine and placebo. The presynaptic α-2 receptors are stimulated by dexmedetomidine, then decreasing norepinephrine release may account for the hypotension and bradycardia.

The incidence of PONV in pediatric patients was reported as high as 34%. However, the incidence in adult appears to decrease with age.^[2,43–45]^ In our meta analysis, the results are consistent with previous study showing that the incidence of PONV in pediatric patients is much higher than that in adult. Operations such as strabismus, adenotonsillectomy may partially explain the higher incidence of PONV in children, although the potential mechanism is complex.

Nausea and vomiting are 2 distinguishing phenomena. Previous report assess the variables independently.^[46]^ However, nausea and vomiting are usually coexistence in a patient, the occurrence of postoperative nausea (PON) or postoperative vomiting (POV) is noticeably parallel to PONV, thus some researches do not try to distinguish the 2 variables. So, we regard the PONV variables as a substitute for PON or POV, if only PON or POV was reported in the trials. Therefore, in this study, we only analyzed the effect of dexmedetomidine on PONV.

Our meta-analysis had some limitations. First, the included studies in different clinical setting would complicate the results of our meta-analysis. Second, prior histories such as motion sickness and nonsmoker were not recorded and analyzed in our study. Third, the different surgical types and length of operation contributed to the heterogeneity in fentanyl consumption. Therefore, more RCTs about this kind of patients and various administration modes of dexmedetomidine during general anesthesia are required to detect the efficacy of dexmedetomidine on PONV.

In conclusion, this current meta-analysis suggested that administration of dexmedetomidine reduce the PONV, also reduced the perioperative fentanyl consumption. Moreover, when we use it in continuous infusion mode, the potential adverse events such as bradycardia and hypotension could reduce.

## Supplementary Material

Supplemental Digital Content
