# Supplementary figures and images for: Dexmedetomidine prevent postoperative nausea and vomiting on patients during general anesthesia: A PRISMA-compliant meta analysis of randomized controlled trials
Source: Medicine (Baltimore). 2017 Jan 10;96(1):e5770. doi: 10.1097/MD.0000000000005770 (PMC5228682; doi:10.1097/MD.0000000000005770)

**supplemental Figure** the effect of dexmedetomidine on children and adult.


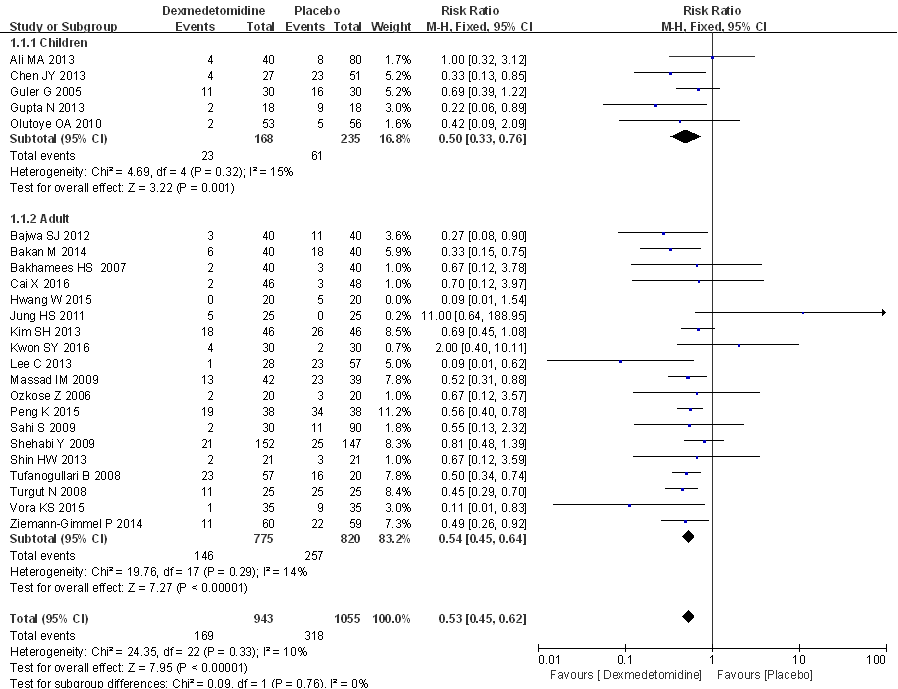

Supplement: Supplemental Digital Content [file medi-96-e5770-s001.doc]
